# Varying (preferred) levels of involvement in treatment decision-making in the intensive care unit before and during the COVID-19 pandemic: a mixed-methods study among relatives

**DOI:** 10.1186/s12911-024-02429-y

**Published:** 2024-02-12

**Authors:** Sophie C. Renckens, H. Roeline Pasman, Zina Jorna, Hanna T. Klop, Chantal du Perron, Lia van Zuylen, Monique A.H. Steegers, Birkitt L. ten Tusscher, Margo M.C. van Mol, Lilian C.M. Vloet, Bregje D. Onwuteaka-Philipsen

**Affiliations:** 1 Department of Public and Occupational Health, Amsterdam UMC, location VU Medical Center, Amsterdam, The Netherlands; 2 Expertise Center for Palliative Care Amsterdam UMC, Amsterdam, The Netherlands; 3 Viaa University of Applied Sciences, Zwolle, The Netherlands; 4 Department of Medical Oncology, Amsterdam UMC, location VU Medical Center, Cancer Center Amsterdam, Amsterdam, The Netherlands; 5 Department of Anaesthesiology, Amsterdam UMC, location VU Medical Center, Amsterdam, The Netherlands; 6 Department of Intensive Care Medicine, Amsterdam UMC, location VU Medical Center, Amsterdam, The Netherlands; 7 Erasmus MC, Department of Intensive Care Medicine Adults, University Medical Center Rotterdam, Rotterdam, The Netherlands; 8 Foundation Family and patient Centered Intensive Care (FCIC), Alkmaar, The Netherlands; 9 Research Department of Emergency and Critical Care, HAN University of Applied Sciences, Nijmegen, The Netherlands; 10 IQ Healthcare, Radboud Institute for Health Sciences, Radboud University Medical Center, Nijmegen, The Netherlands

**Keywords:** ICU, Family support, Decision-making, Quality of life, COVID-19, Critical care

## Abstract

**Background:**

In the intensive care unit (ICU) relatives play a crucial role as surrogate decision-makers, since most patients cannot communicate due to their illness and treatment. Their level of involvement in decision-making can affect their psychological well-being. During the COVID-19 pandemic, relatives’ involvement probably changed. We aim to investigate relatives’ involvement in decision-making in the ICU before and during the pandemic and their experiences and preferences in this regard.

**Methods:**

A mixed-methods study among relatives of ICU patients admitted to an ICU before or during the COVID-19 pandemic. Relatives in six ICUs completed a questionnaire (*n* = 329), including two items on decision-making. These were analysed using descriptive statistics and logistic regression analyses. Subsequently, relatives (*n* = 24) were interviewed about their experiences and preferences regarding decision-making. Thematic analysis was used for analysing the qualitative data.

**Results:**

Nearly 55% of the relatives indicated they were at least occasionally asked to be involved in important treatment decisions and of these relatives 97.1% reported to have had enough time to discuss questions and concerns when important decisions were to be made. During the first COVID-19 wave relatives were significantly less likely to be involved in decision-making compared to relatives from pre-COVID-19. The interviews showed that involvement varied from being informed about an already made decision to deliberation about the best treatment option. Preferences for involvement also varied, with some relatives preferring no involvement due to a perceived lack of expertise and others preferring an active role as the patient’s advocate. Discussing a patient’s quality of life was appreciated by relatives, and according to relatives healthcare professionals also found this valuable. In some cases the preferred and actual involvement was in discordance, preferring either a larger or a smaller role.

**Conclusions:**

As treatment alignment with a patient’s values and preferences and accordance between preferred and actual involvement in decision-making is very important, we suggest that the treatment decision-making process should start with discussions about a patient’s quality of life, followed by tailoring the process to relatives’ preferences as much as possible. Healthcare professionals should be aware of relatives’ heterogeneous and possibly changing preferences regarding the decision-making process.

**Supplementary Information:**

The online version contains supplementary material available at 10.1186/s12911-024-02429-y.

## Background

Most patients in the intensive care unit (ICU) are unable to communicate and participate in decision-making due to the severity of their illness, invasive mechanical ventilation, delirium and/or sedation [1]. Consequently, close relatives have a pivotal role as a patient’s surrogate decision-maker in the ICU [2]. Research shows that relatives have varying preferences as to what extent they wish to be involved in decision-making [3–6]. In addition, physicians can adopt roughly two approaches, namely physician-driven or shared decision-making [7]. In a physician-driven approach, also known as a paternalistic approach, physicians announce treatment decisions to relatives and point out that decision-making is a medical responsibility, leaving little room for relatives to be involved. Physicians that adopt a shared approach generally stress that decision-making is a collaborative process between relatives and physicians, based on the patient’s preferences and conception of quality of life. Physicians can also switch back and forth between these approaches, as well as combining aspects of the two approaches. Many physicians do not verify whether their decision-making approach matches with relatives’ preferences [7]. This could result in discordance between the preferred and actual involvement of relatives in decision-making, which in turn was found to be associated with more symptoms of post-traumatic stress disorder (PTSD) and depression among relatives [8]. In contrast, if the actual involvement is in accordance with the preferred involvement, relatives’ acceptance of and coping with the (outcome) of treatment decisions is likely to be enhanced.

Involving relatives in decision-making in the ICU might have been endangered during the COVID-19 pandemic as the ICU’s daily practice was significantly changed. Firstly, during the first few months of the pandemic, ICUs relied almost exclusively on telecommunication in their communication with relatives [9, 10]. Secondly, in some ICUs so-called family support teams, consisting of non-ICU medical specialists, took over the supporting role for relatives from ICU healthcare professionals [11–13]. This altered support practice in the ICU might have led to changes in the extent to which relatives were involved in decision-making. Ramos et al. [14] showed that during the pandemic relatives who received information for decision-making by video call evaluated the inclusion of relatives in decision-making, as well as the quality of information more negatively compared to relatives who received information face-to-face. Additionally, in the early stages of the pandemic a lot was unknown about the treatment of a COVID-19 infection, which possibly also impacted the treatment decision-making process.

Since discordance between preferred and actual involvement of relatives in decision-making can have negative consequences for the relatives, which might have been exacerbated during the pandemic, it is important to gain more insight into the experiences of relatives with involvement in treatment decision-making in the ICU. This is especially important for healthcare professionals who can adapt their practice if needed. To our knowledge, little is known about relatives’ involvement in treatment decision-making in the ICU during the pandemic, and how this possibly differs from the situation before the pandemic. Therefore in this study we aim to examine the level of involvement of relatives in decision-making in the ICU before and during the COVID-19 pandemic and explore relatives’ experiences and wishes in this regard. The differences and similarities in (experiences with) involvement in treatment decision-making in the ICU before and during the pandemic, can provide insights into how to best approach treatment decision-making in future care under normal circumstances and in case of a new pandemic.

## Methods

### Design

We conducted a sequential explanatory mixed-methods study. First, we administered a retrospective cross-sectional questionnaire among first contact persons of ICU patients from three different time periods (pre-COVID-19, first COVID-19 wave and second COVID-19 wave, see Table 1). Subsequently, qualitative data were acquired through in-depth semi-structured interviews with first contact persons of ICU patients from two time periods (pre-COVID-19 and first COVID-19 wave). The coherence between the two methods was twofold. First, on a sample level results from the quantitative questionnaire were used to inform the development of the topic guide and subsequently the follow-up interviews were used to gain an in-depth understanding of certain findings from the questionnaire. Second, on an individual level the individual questionnaire results were used to purposively sample relatives for the interviews, as well as to personalize, to some extent, the individual interviews. The current study is part of a larger mixed-methods study in which also other topics were covered such as important elements of support [26], well-being of relatives and support for relatives in the period around the end-of-life of a patient.

### Decision-making procedures in ICUs in the Netherlands

Since January 2020, the principle of shared decision-making is enshrined in the Dutch law on the medical treatment agreement (WGBO). In the law is it now explicitly mentioned that shared decision-making is a prerequisite during doctor-patient encounters (Article 7a: 448 Dutch Civil Code) [15]. This entails explicit exploration of the patient’s wishes, views and preferences and their integration in medical decision-making [16]. This applies to doctor-patient encounters in general, so also in ICUs. However, most patients in the ICU are incompetent and therefore have or get appointed a representative (Article 7a: 465 Dutch Civil Code) [15]. In the ICU, physicians should thus in many cases involve the patient’s representative in decision-making rather than the patient himself. The national guideline *palliative care and abstaining from life-prolonging treatments in adult ICU patients* of the Dutch Association for Intensive Care (NVIC) mentions that the decision to withhold or withdraw treatment is in principle made by the treating ICU physician in consultation with other physicians who are involved in the patient’s treatment and the patient or the patient’s representatives [17]. A decision to withhold or withdraw treatment by a representative of an incompetent patient does not automatically have the same status as the decision of the competent patient himself and must be tested against the patient’s best interests, the patient’s presumed will and good patient’s representation (not serving other interests). According to the guideline a request by a patient’s representative to withhold or withdraw treatment should be considered, but need not be followed, especially when the physician does not consider treatment to be futile or undesirable. Similarly, a patient’s representative cannot block the decision to withhold or withdraw treatment, if this decision is made by physicians on medical grounds, but seeking consent is of great importance [17].

### Study population and data collection

The study was performed in six Dutch ICUs in the Northwestern part of the Netherlands. Two of the six ICUs were located in two affiliated academic hospitals, and the other four were general hospitals. During the first COVID-19 wave three of the six ICUs used newly developed family support teams (Supplementary file 1) that supported relatives via telecommunication, whereas in the other three ICUs the ICU healthcare professionals continued providing the support, yet also via telecommunication.

### Quantitative questionnaire study

First contact persons of ICU patients were eligible if the patient had all characteristics as listed in the in-and exclusion criteria in Table 1, and first contact persons themselves had sufficient proficiency in the Dutch language. There was one first contact person per patient. All first contact persons in the participating six Dutch ICUs were eligible and no selection was made in this.

**Table 1:** In- and exclusion criteria

Inclusion criteria First contact person of an intensive care unit (ICU) patient with the following inclusion criteria:			
	**Pre-COVID-19**	**First COVID-19 wave**	**Second COVID-19 wave**
**Age patient**	≥ 18 years	≥ 18 years	≥ 18 years
**Period of ICU stay**	December 1, 2019– February 1, 2020	March 15– May 15 2020	October 1, 2020– January 1, 2021
**Length of ICU stay**	≥ 3 days	≥ 3 days	≥ 3 days
**Diagnosis of the patient**	N/A	Confirmed COVID-19 infection	Confirmed COVID-19 infection
**Other criteria regarding patient**	Invasive mechanical ventilation ≥ 3 days^1^	N/A	N/A
Exclusion criteria			
The first contact person has insufficient proficiency in the Dutch language			

N/A = not applicable

1 This criterion was chosen for comparability with the COVID-19 periods in which the majority of COVID-19 patients in the ICU had invasive mechanical ventilation.

**Fig. 1:**
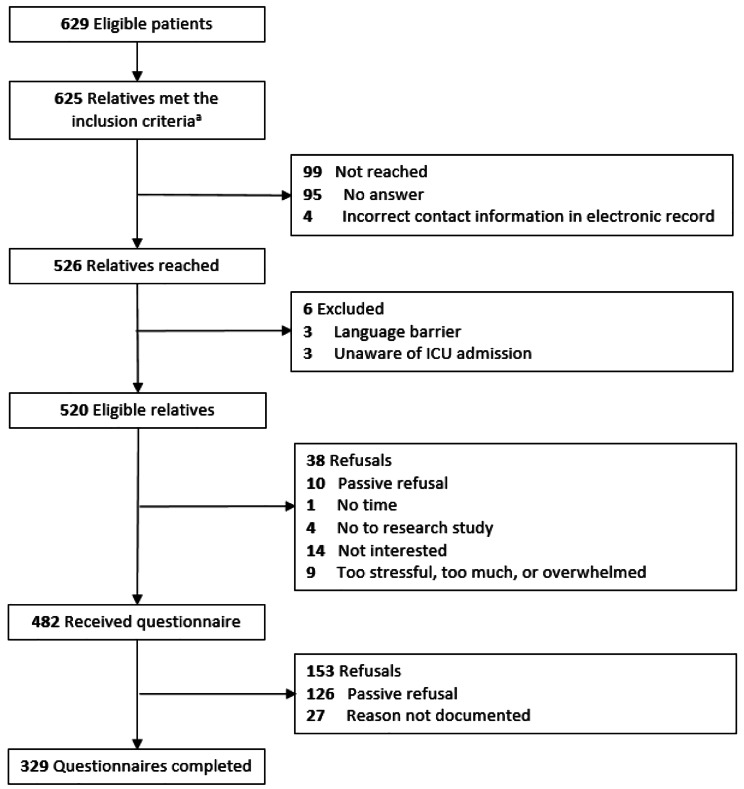
Eligibility and enrolment flowchart. ^a^Four relatives were the first contact person for two patients in the intensive care unit

Medical records of ICUs were automatically searched for eligible patients using a standard query created by one researcher (CdP), following an additional manual eligibility check by two researchers (CdP and SCR). If the patient was eligible, the contact information of the first contact person was abstracted. Two researchers (CdP and SCR) approached these first contact persons by telephone between January and July 2021 for participation in the questionnaire study. The median time between a patient’s ICU admission date and study recruitment was 9.2 months (range 4–18 months) A maximum of three attempts to reach a relative by telephone were made, in which study information was provided and a short eligibility check was performed. If relatives provided oral consent to receive the study information and written questionnaire by mail, this was sent to them within 7 days. Relatives were asked to consent to study participation at the start of the written questionnaire. Relatives were sent reminder letters after three and six weeks if they had not yet responded. A total of 625 relatives were found eligible for participation in the questionnaire study, of whom 526 were reached by telephone (Fig. 1). Six of the 526 relatives were excluded due to a language barrier or being unaware of the ICU admission. Of the 520 eligible relatives, 329 relatives returned a completed questionnaire (response 63%).

### Qualitative interview study

At the end of the questionnaire, relatives were asked if they gave consent to be contacted about participation in an interview. In total 164 relatives from pre-COVID-19 and the first wave gave their consent. Relatives were selected through purposive sampling. As much variation as possible was sought with regard to kinship to the patient; gender of the relative; patient deceased or not; ICU location and being supported by a family support team or not. We phoned 34 relatives to ask if they were willing to participate in an interview. When the relative agreed, a date and time were set. Of the 34 relatives who were approached, 30 participated. The four relatives that did not participate were unavailable by phone. In six interviews the subject of the present study, decision-making, was not discussed due to time constraints. Therefore data from 24 interviews have been used in analyses.

Interviews were conducted between May 5, 2021, and September 28, 2021, by four researchers. Twelve interviews were conducted by one researcher (SCR), eight by a second researcher (ZJ), two by a third researcher (HTK) and two by a fourth researcher (CdP). All interviewers had received training in conducting qualitative interviews and had no relation to the participating relatives. The average duration of the interviews was 45 min (15–65 min). Because of the COVID-19 restrictions, all interviews were conducted by telephone (*n* = 20) or via video call (*n* = 4), according to relatives’ preference. All interviews were audio-recorded and transcribed verbatim.

### Measurements

#### Quantitative questionnaire study

The questionnaire included a wide range of questions regarding the experiences of relatives with the support they received during the patient’s ICU admission and their well-being (Supplementary file 2). The variables that are of interest to the present study are the relatives’ and patients’ demographic characteristics and two items on relatives’ experiences with decision-making in the ICU (Supplementary file 2, question 40 and 41).

Data on gender and whether the patient died in the ICU were abstracted from the patient’s medical record. Additionally, in the questionnaire relatives were asked about kinship, age, gender, level of education and cultural background of the relative and age of the patient. The two items on relatives’ experiences with decision-making were: (1) were you asked to be involved in important treatment decisions?; (2) was there enough time to discuss your concerns and questions when important decisions were to be made? Both items could be scored as never, occasionally, usually, always or not applicable.

#### Qualitative interview study

The interviews were semi-structured and used a topic list (Supplementary file 3). Some questions were based on particular individual survey responses (e.g. “In the questionnaire you mentioned that you were sometimes asked to be involved in important treatment decisions. Could you tell a bit more about this?”). The topics from the interviews that will be discussed in this study are relatives’ experiences with decision-making in the ICU and their preferences in this regard.

### Analysis

#### Quantitative questionnaire study

Sample characteristics were analysed using descriptive statistics, both for the total population and for relatives from the three time periods separately. The two items on decision-making in the ICU were also analysed using descriptive statistics. To analyse potential differences in these two items between relatives from the three time periods the two items were dichotomised. Item 1 was dichotomised into never and occasionally/often/always, indicating whether they were to any extent involved in decision-making. Item 2 was dichotomised into never/occasionally and often/always, indicating to what extent there was enough time to discuss questions and concerns. Data on item 2 were only analysed for relatives who answered that they were occasionally, often or always involved in decision-making (item 1). For both items the answering option not applicable was treated as a missing value. Differences in the two items between the three COVID-19 periods were analysed using binary logistic regression analyses corrected for confounders (> 10% change of the regression coefficient) and/or effect modifiers (significant interaction term), and a *p*-value of < 0.05 was considered significant. Several variables were tested for confounding and/or effect modification: time between ICU admission date and questionnaire completion, relative’s gender, age, level of education and cultural background, and whether the patient was deceased or not. For both items 1 and 2, the time between admission and questionnaire completion and level of education were confounders. In addition, cultural background was also a confounder for item 2. Similarly, differences between relatives from the first COVID-19 wave who primarily received support from a family support team and relatives from the first COVID-19 wave who received support from ICU healthcare professionals were analysed.

#### Qualitative interview study

The interviews were analysed following the principles of thematic analysis [18]. First, the researchers familiarised themselves with the data by reading the transcripts thoroughly. Next, interviews were coded inductively using MAXQDA 2020. The first three interviews were independently coded by two researchers (SCR and ZJ), and then extensively discussed by four researchers (SCR, ZJ, HRP and BOP). Based on this discussion some codes were refined. Subsequently, the aforementioned procedure was repeated with two new interviews. This resulted in a refined code book, which was used by one researcher (SCR) to code the remaining 19 interviews. The initial codes were collated into overarching themes by the research team. After 24 interviews were coded, we concluded that no additional interviews were needed because no longer any new themes emerged. The themes were discussed and grouped within the research team. Finally, quotes were translated by a professional translator and checked by a second professional translator. The research group consisted of researchers with different backgrounds (health sciences, medical anthropology, sociology, social psychology), as well as both physicians and nurses.

### Ethics

Relatives were informed about the questionnaire and interview both orally and in writing. Before filling in the questionnaire, relatives provided written informed consent. Before the interview, relatives gave oral informed consent. After transcription, the audio recordings were deleted and the transcript were anonymised to ensure participants’ privacy. The Medical Ethics Review Committee of VU University Medical Center determined exception from formal review under Dutch law (registration number 2020.0618). Additionally, institutional review boards at each site approved all procedures (Dijklander Science Centre and Board of Directors Dijklander Ziekenhuis (DOC 020), Board of Directors Ziekenhuis Amstelland (n.s.), Board of Directors Zaans Medisch Centrum (HF21038), Science Office Noordwest Ziekenhuisgroep (L021-037)).

## Results

### Participants

The 329 participating relatives in the questionnaire were mostly the partner of the patient (52.3%), women (71.6%), 51 years or older (65.3%), medium or highly educated (80.9%), and had a Dutch cultural background (91.5%) (Table 2). The admitted patients were mostly men (67.2%), 66 years or older (49.2%) and stayed at the ICU for 11 days or longer (62.9%). In total 27.4% of the patients deceased during their ICU admission. During the first and second wave patients were significantly more often men (resp. 73.1% and 69.2%) compared to pre-COVID-19 (56.8%), whereas relatives were more often women (resp. 76.0% and 74.8% compared to 62.1%). Relatives from the second wave were significantly less often from a Dutch cultural background, and more often from another cultural background (e.g. Surinamese or Moroccan). In addition, non-response analysis showed that relatives who completed the questionnaire were more often the partner (52.3%) and less often the child of the patient (31.9%) compared to relatives who did not complete the questionnaire (respectively 34.1% and 45.3%). There were no statistically significant differences in gender of the patient and whether the patient had deceased or not between relatives who did and who did not participate.

The 24 relatives that were interviewed were mainly female (*n* = 16), partner of the patient (*n* = 14) or their child (*n* = 9), and from the first COVID-19 wave (*n* = 17) (Table 3). The loved one of 10 relatives deceased in the ICU.

**Table 2:** Relative and patient demographic characteristics from the questionnaire (absolute numbers and rounded percentages)

		Pre-COVID-19 (*n* = 95)	First COVID-19 wave (*n* = 130)	Second COVID-19 wave (*n* = 104)	Total (*n* = 329)	*p*-value
*Relative characteristics*						
Bereaved relative		25 (26.3)	39 (30.0)	26 (25.0)	90 (27.4)	0.692
Kinship to patient						0.574
	Partner	49 (51.6)	72 (55.4)	51 (49.0)	172 (52.3)	
	Child	27 (28.4)	41 (31.5)	37 (35.6)	105 (31.9)	
	Other	19 (20.0)	17 (13.1)	16 (15.4)	52 (15.8)	
Gender						**0.047**
	Man	36 (37.9)	31 (24.0)	25 (24.3)	92 (28.1)	
	Woman	59 (62.1)	98 (76.0)	77 (74.8)	234 (71.6)	
	Other	0 (0)	0 (0)	1 (1.0)	1 (0.3)	
Age						0.117
	< 30 years	2 (2.1)	5 (3.8)	6 (5.8)	13 (4.0)	
	30–50 years	25 (26.3)	38 (29.2)	38 (36.5)	101 (30.7)	
	51–65 years	38 (40.0)	56 (43.1)	45 (43.3)	139 (42.2)	
	66 years or older	30 (31.6)	31 (23.8)	15 (14.4)	76 (23.1)	
Level of education						0.088
	Low	16 (17.0)	26 (20.5)	20 (19.4)	62 (19.2)	
	Medium	44 (46.8)	50 (39.4)	54 (52.4)	148 (45.7)	
	High	34 (36.2)	51 (40.2)	29 (28.2)	114 (35.2)	
Cultural background*						
	Dutch	91 (95.8)	124 (95.4)	86 (82.7)	301 (91.5)	**0.001**
	Other^a^	5 (5.3)	12 (9.2)	22 (21.2)	39 (11.9)	**0.002**
COVID-19 during ICU admission		0^b^	54 (42.2)	39 (37.5)	93 (40.1)^c^	0.535
*Patient characteristics*						
Gender						**0.011**
	Man	54 (56.8)	95 (73.1)	72 (69.2)	221 (67.2)	
	Woman	41 (43.2)	32 (24.6)	32 (30.8)	105 (31.9)	
Age						0.152
	< 30 years	1 (1.1)	1 (0.8)	2 (1.9)	4 (1.2)	
	30–50 years	7 (7.4)	12 (9.2)	11 (10.6)	30 (9.1)	
	51–65 years	36 (37.9)	52 (40.0)	45 (43.3)	133 (40.4)	
	66 years or older	51 (53.7)	65 (50.0)	46 (44.2)	162 (49.2)	
ICU length of stay						0.248
	3–4 days	7 (7.4)	5 (3.8)	2 (1.9)	14 (4.3)	
	5–10 days	34 (35.8)	36 (27.7)	38 (36.5)	108 (32.8)	
	11–20 days	25 (26.3)	46 (35.4)	37 (35.6)	108 (32.8)	
	> 20 days	29 (30.5)	43 (33.1)	27 (26.0)	99 (30.1)	

^a^ e.g. Surinamese and Moroccan

^b^ value assumed because of pre-COVID-19

^c^ total calculated based on groups with valid data on this variable

*: multiple answers possible

Missing values: gender relative 2, education 5, COVID-19 2, gender patient 3

**Table 3:** Characteristics of relatives participating in interviews

	COVID-19 period	Gender	Kinship	Gender patient	Age patient	Family support team	Patient died in ICU
1	First wave	Female	Child^a^	Male	51–65 years	Yes	Yes
2	First wave	Female	Partner	Male	66–80 years	Yes	Yes
3	First wave	Female	Partner	Male	30–50 years	Yes	No
4	Pre-COVID-19	Female	Partner	Male	51–65 years	N/A	No
5	First wave	Male	Child^a^	Female	66–80 years	Yes	Yes
6	Pre-COVID-19	Male	Child^a^	Female	66–80 years	N/A	No
7	First wave	Female	Partner	Male	51–65 years	Yes	No
8	First wave	Female	Child^a^	Female	66–80 years	No	Yes
9	First wave	Male	Partner	Female	66–80 years	Yes	Yes
10	Pre-COVID-19	Female	Child^a^	Male	80 + years	N/A	Yes
11	First wave	Female	Child^a^	Female	66–80 years	Yes	No
12	First wave	Female	Child^a^	Male	66–80 years	No	No
13	First wave	Female	Partner	Male	51–65 years	No	No
14	First wave	Female	Partner	Male	51–65 years	Yes	No
15	Pre-COVID-19	Male	Partner	Female	66–80 years	N/a	Yes
16	First wave	Female	Partner	Male	51–65 years	Yes	No
17	First wave	Female	Partner	Male	51–65 years	No	No
18	Pre-COVID-19	Female	Child^a^	Female	66–80 years	N/A	No
19	Pre-COVID-19	Male	Partner	Female	80 + years	N/A	No
20	First wave	Female	Partner	Male	51–65 years	Yes	Yes
21	First wave	Male	Child^a^	Female	51–65 years	No	No
22	First wave	Male	Parent	Male	30–50 years	Yes	No
23	Pre-COVID-19	Female	Partner & child^a,b^	Male	51–65 years	N/A	Yes
24	Pre-COVID-19	Male	Partner	Female	51–65 years	N/A	Yes

^a^ Children in law are also included in this category

^b^ Interview with both the partner and the child of a patient

### Varying levels of involvement in treatment decision-making

Across relatives from the questionnaire in all time periods, 21.7% indicated they were always asked to be involved in important treatment decisions, 13.0% often, 19.3% occasionally and 28.0% never (Table 4). In total, 18.0% of the relatives said that the question was not applicable. In the interviews, relatives explained what they considered important treatment decisions and this included amongst others: whether a patient will receive invasive mechanical ventilation, use of particular medication, participation in clinical trials or experimental therapy, and how long to continue treatment considering the quality of life. Of relatives who were to any extent involved in important treatment decisions, 59.2% reported that there was always enough time to discuss their questions and concerns, 25.3% often, 12.6% occasionally and 1.1% never.

**Table 4:** Relatives’ involvement in decision-making (absolute numbers and rounded percentages)

		Pre-COVID-19 N (%)	First COVID-19 wave N (%)	Second COVID-19 wave N (%)	Total N (%)
Asked to be involved in treatment decisions		*N* = 95	*N* = 130	*N* = 104	*N* = 329
	Always	24 (26.4)	27 (20.9)	19 (18.6)	70 (21.7)
	Often	12 (13.2)	15 (11.6)	15 (14.7)	42 (13.0)
	Occasionally	17 (18.7)	21 (16.3)	24 (23.5)	62 (19.3)
	Never	16 (17.6)	46 (35.7)	28 (27.5)	90 (28.0)
	Not applicable	22 (24.2)	20 (15.5)	16 (15.7)	58 (18.0)
Enough time for questions and concerns with treatment decisions^a^		*N* = 53	*N* = 63	*N* = 58	*N* = 174
	Always	36 (67.9)	37 (58.7)	30 (51.7)	103 (59.2)
	Often	11 (20.8)	14 (22.2)	19 (32.8)	44 (25.3)
	Occasionally	5 (9.4)	10 (15.9)	7 (12.1)	22 (12.6)
	Never	0	1 (1.6)	1 (1.7)	2 (1.1)
	Not applicable	1 (1.9)	1 (1.6)	1 (1.7)	3 (1.7)

^a^ Only included for relatives who were always/often/occasionally asked to be involved in treatment decisions

**Table 5:** Logistic regression: differences in involvement in treatment decisions between COVID-19 periods

	Asked to be involved in treatment decisions		Enough time for questions and concerns with treatment decisions	
	Row %^a^	Adjusted OR (95% CI)^b^	Row %^c^	Adjusted OR (95% CI)^d^
Pre-COVID-19	76.8	1.00	90.4	1.00
First COVID-19 wave	57.8	0.41 (0.17–0.98)	82.3	0.28 (0.07–1.19)
Second COVID-19 wave	67.4	0.63 (0.16–2.43)	86.0	0.24 (0.03–2.28)

^a^ Percentage of the relatives who reported to have been always, often or occasionally involved in treatment decisions;

^b^ Adjusted for the period between ICU admission and questionnaire completion and level of education;

^c^ Percentage of relatives who reported to have had always or often enough time for questions and concerns with treatment decisions;

^d^ Adjusted for the period between ICU admission and questionnaire completion, level of education and cultural background;

Table 5 shows the results of logistic regression analyses that were used to assess if there was a difference in decision-making involvement for relatives from the three COVID-19 periods. The likelihood that relatives were asked to be involved in important treatment decisions was lower for relatives from the first COVID-19 wave as compared to relatives from pre-COVID-19 (OR 0.41). Although not supported by sufficient statistical evidence, the point estimate in combination with the 95% CI (OR 0.28 (0.07–1.19)) suggests that relatives from the first COVID-19 wave were also less likely to have had enough time to discuss questions and concerns with treatment decisions compared to relatives from pre-COVID-19. The likelihood that relatives from the second wave were asked to be involved in treatment decisions or had enough time for questions and concerns did not significantly differ from relatives from pre-COVID-19.

In the questionnaire results no significant differences were found in involvement in treatment decisions between relatives from the first COVID-19 wave who were supported by the ICU (64.3%) or those who were supported by a family support team (55.6%) (Supplementary file 1). Neither was there a significant difference in the percentage of relatives who indicated there was enough time to discuss questions and concerns (resp. 83.3% and 81.8%) (Supplementary file 1).

Findings from the interviews with relatives from pre-COVID-19 and the first wave showed that there is a heterogeneity in experiences and wishes regarding involvement in decision-making in the ICU. As in the questionnaire, relatives reported various levels of involvement, and it varied as to what relatives considered “being involved”. Several interviewees indicated that they were never involved in decision-making in the ICU. They described that they were informed by a physician about certain treatment decisions that were already made and executed and therefore relatives had no more influence on the decision. As two relatives explained about the decision to intubate their loved one:

*“Well, look, that intubation wasn’t really a decision moment; they just went ahead and did that. Right, you just get told that.”* (14: partner, female, patient discharged from ICU, first wave).

*“What it comes down to is that he was put into a coma within one night because he was deteriorating really fast. And that wasn’t really discussed with us. We weren’t able to say goodbye to my father either because he was in such a bad state that the doctors decided to put him in a coma first and only then tell us about it.”* (1: child, female, patient died in the ICU, first wave).

One relative explained that her husband was administered a drug without her knowledge that resulted in unpleasant side effects:

*“No, I didn’t know anything about it. He only told me later that he’d been given that medication [chloroquine] and it had given him hallucinations.”* (13: partner, female, patient discharged from ICU, first wave).

The interviewees that indicated that they were involved in decision-making mentioned different levels of involvement. Some relatives were contacted by a physician about an important treatment decision that was to be executed, but they did not feel they could participate in a discussion about this decision. In these instances, physicians informed relatives about the treatment decision they had in mind but relatives did not feel invited to ask questions or share their thoughts.

*“Not having an input in the decisions. We did in the nursing care, bit by bit, and how they could get my mother to the stage where she pushes herself again or whether she does this or does that. […] But that really complicated stuff, things where wecouldn’thave taken a decision, well no, other than them explaining it. But not them asking: what do you think about this?”* (18: child, female, patient discharged from ICU, pre-COVID-19).

One relative indicated that he was never invited to treatment decision discussions, except for the end-of-life decision.

*“At first, you’re not allowed there at all, at first, you’re not allowed to do anything and then all of a sudden it’s, well, the end, and you know it’s over and all of a sudden you have to come and say… At first, you don’t have any say at all, because they all decide exactly what needs to be done. Which is fine, that’s what they’re experts in, but then all of a sudden you have to come and say what decision needs to be taken. Then you yourself have to come and take the decision; that’s how I’d put it.”* (9: partner, male, patient deceased in ICU, first wave).

Several other relatives mentioned that they were actively involved in decision-making, which they explained as being asked to provide consent for several treatments and/or being asked what they thought about a proposed treatment decision.

*“Then I got a phone call Monday morning saying we’re going to sedate her because otherwise she’ll choke on her own mucus. Then she was put on the ventilator. […] I had to give permission for them to do that, of course. Because it wasn’t a case of we’re going to put her to sleep and this is the situation…”* (15: partner, male, patient deceased in ICU, pre-COVID-19).

Another level of involvement that was mentioned by the interviewed relatives was that, before important treatment decisions had to be made, they were asked to explain what quality of life would entail for their loved one and what their wishes were. This information was then used by physicians to inform decision-making about how long to continue treatment.

*“And in the weekend before [the death], I spoke to a female ICU specialist who’d just had a chat with me about how she was going to have this talk and I should start thinking about what the quality of life was. And so they came back on that later in the week. So I told them what I thought the quality of life was for [patient’s name]. Afterwards, they gave feedback saying it was incredibly important that they’d heard in such detail what kind of a person they were dealing with. And it had helped them hugely in taking the decision.”* (2: partner, female, patient deceased in ICU, first wave).

### Preferences and feelings regarding involvement in treatment decision-making

Relatives who were not or limited involved in treatment decision-making differed as to what extent they wished to have been involved. Some of these relatives strongly wished to have been (more) involved in decision-making whereas others were fine with their low level of involvement. Relatives who expressed a wish to have been (more) involved in decision-making explained that the absence of involvement in treatment decision-making led to feelings of powerlessness and dependency on healthcare professionals. One relative mentioned that she wanted to ask questions about the treatment and provide possible relevant information about the patient:

*“Well, just that feeling that there’s nothing you can do. We really wanted to drive and run over to the hospital and corner one of those doctors to ask them questions and tell them things. But you can’t do anything.”* (1: child, female, patient died in ICU, first wave).

Relatives described two main reasons for their strong desire to have been (more) involved in treatment decision-making during the ICU admission of their loved ones. On the one hand, they felt that in case they would have been more involved they would be better able to judge whether the right decisions were made or not. By being left out of the decision-making, relatives felt they lacked important information to do so.

*“What I personally felt really very strongly was that there was this strong focus on the medical complaint she has but they didn’t really take enough account of her complete medical history. […] That’s really the only point that I had real difficulty with, the feeling from day one… not that I was being ignored, but that the focus was very much on this specific aspect and that recovery, which made me think: the way you lot are going about it now, I’m afraid that recovery can never happen and it’s not realistic given her medical history. And that’s the essence of what I had the most difficulty with, that I wanted a dialogue with the doctor about the period afterwards, the recovery period. And whether this was actually the right approach. Not that I had doubts about their approach, but I did want to know: have you taken everything into account in that assessment?”* (6: child, male, patient discharged from ICU, pre-COVID-19).

On the other hand, relatives indicated that they felt that they had very important information about the wishes and preferences of their loved one, which they wished was used by healthcare professionals to inform important treatment decisions.

*“Well, we were like, what happens when you take him off the ventilator? Then he’ll die. So does it make sense to carry on? How long do you go on for? And they were like, well, we’ll carry on for three weeks at any rate because we… And that was the story, we’d heard from China– that’s how it went, of course– that people can get better after three weeks. […] I’ve got another point for improvement. Ask the question in advance about how far you want to go. That was a recurring theme throughout. […] He would definitely not have wanted to wake up based on what he… I really think he wouldn’t have wanted that. And then you carry on because China says you’ll get through it after three weeks… but how? Of course, a lot of things were unclear at that point but perhaps you should first discuss things with the family, so this is indeed unclear, we don’t know how long it can go on and still have the possibility of him waking up. […] What is the guiding principle and what can you get at the end? I mean, what are you like when you come out of it? That was an important aspect we were always worrying about. […] Because we knew he’d been very clear about that [not wanting to become a vegetable].”* (12: child, female, patient discharged from ICU, first wave).

As indicated previously, several relatives who were not or to a limited extent involved in decision-making were fine with their limited role. They mentioned two main reasons for this. First, some interviewed relatives indicated that the situation of their loved one in the ICU was often so acute that there would have been no possibility at all for healthcare providers to consult them in these treatment decisions. Second, relatives explained that they felt that they did not have the expertise to participate in decision-making and that physicians are far more knowledgeable to make treatment decisions. For example, relatives mentioned that they know little or nothing about the equipment and drugs used in the ICU.

*“Right, those are all decisions they have to take there on the spot, often in short order. […] Well, they basically take all the decisions. And you only really hear about them later. Because you don’t really have any say in them, as it were. And I actually think that’s a good thing, because they’re the ones who know about these things, not me. […] So I don’t really mind because it’s exactly what I said: stick to what you know– and they’re the people who know about these things, not me.”* (17: partner, female, patient discharged from ICU, first wave).

Lacking expertise was also mentioned by relatives who indicated that they were involved in decision-making but did not feel a need to be involved.

*“But they just told us that: this is what we’re planning and what do you think? Well, you lot are the specialists. That was kind of how it went. I reckon there comes a point when you’ve spoken to the doctor a few times, and by then they have it figured.”* (21: child, male, patient discharged from ICU, first wave).

This theme of being fine with no involvement because of a perceived lack of expertise seemed to be more prominent in the interviews with relatives from the first COVID-19 wave than in the interviews with relatives from pre-COVID-19.

Relatives who felt that they had a say in certain treatment decisions experienced a variety of feelings about this. These included both appreciative and burdensome feelings, as well as a combination of the two. A few relatives indicated that they felt it was a very big responsibility to make a decision in which the life of a loved one is at stake and that this was emotionally burdensome. One relative explained that she was asked to decide for her partner with a COVID-19 infection about participation in a clinical trial with an experimental drug. According to this relative, the physician told her that it was a decision about life or death. She experienced it as a very hard decision to make:

I: *“What is it that makes it so difficult: that it has to be done so quickly?”*

*R: “Yes, but it’s also because you don’t know anything about that disease. I was sitting watching television and then I saw: number 100 is dead, 101, 102 is dead. And so on. He was lying there and was still alive, so I wanted to keep it that way. So yes, it’s really difficult to make a choice because you don’t know. You don’t know what the medicine will do because it was in the test phase. […] Right, I found it incredibly difficult because, well, you’re deciding about someone else’s life. Look, if you were having to choose for yourself, the choice would be a bit easier.”* (17: partner, female, patient discharged from ICU, first wave).

A few relatives mentioned that their involvement in decision-making made them accept the situation and the outcome of the treatment more easily.

*“The nice thing is that because you get so involved, you’re also… well, I wouldn’t say you’re at peace with it, but you do accept it.”* (5: child, male, patient deceased in ICU, first wave).

Finally, some relatives who indicated that they were, to some extent, involved in decision-making expressed satisfaction with the support they received during this decision-making process. They mentioned that there were possibilities to discuss their questions and concerns and that the healthcare professionals gave them time to think about certain decisions.

*“So there was the conversation we had Monday evening with the doctor and the nursing staff. Of course that was very confronting because then you have to decide: OK, are we going to resuscitate him one more time, or are we going to say no, it’s fine this way, and if he continues to struggle, we’re not going to do it again? And I think they guided us very well because I had reached that stage, but my brother hadn’t at that point. So then they started again… because there were the three of us– my husband, my brother and me. We had that conversation, then they left us for a bit, saying we’re going to leave the three of you to think about this situation and right, what we should do now.”* (10: child, female, patient deceased in ICU, pre-COVID-19).

## Discussion

Both the questionnaire and interview results showed that the level of involvement in treatment decision-making in the ICU varied substantially among relatives. This was the case both within and between the study periods. Also, relatives from the first COVID-19 wave were significantly less likely to be involved compared to relatives from pre-COVID-19. Additionally, relatives expressed different understandings of the concept of “involvement”, varying from being informed about a decision that was already made to discussing with healthcare professionals about the best treatment option. Finally, diverse preferences and feelings regarding involvement in decision-making were found among relatives. Some relatives preferred a limited role in decision-making mainly due to a perceived lack of expertise. Others wished to be actively involved because they considered themselves the best patient’s advocate. Several relatives who were involved described that this was emotionally hard, but some were also appreciative of it. For a part of the relatives, their actual and preferred role in treatment decision-making was in accordance, whereas there were also relatives who preferred to have had a smaller role and relatives who wishes to have been more actively involved.

### Decision-making ranging from physician-driven to shared

Nearly 30% of the relatives in our study indicated that they were never involved in treatment decision-making, while a little more than 30% reported being always or often involved. Important to highlight is that the interviews revealed that relatives had different interpretations of what active involvement in decision-making in the ICU looked like. While some relatives considered being asked to provide consent for a certain treatment decision as active involvement, others did not find it sufficient to speak about active involvement. Akkermans et al. [7] described that the involvement of relatives in decision-making is actually a continuum ranging from a physician-driven approach to a fully shared decision-making approach, which was also reflected in our sample. Based on our results, it appears that in many cases physicians adopt the physician-driven approach or are somewhere in the middle of the continuum, making the important treatment decision without or limitedly involving relatives in this process. Existing evidence suggests that one of the reasons why physicians appear hesitant to give relatives some control in decision-making is a lack of trust that relatives can understand a decision and its consequences [19]. In addition, physicians fear that too much involvement of relatives will lead to more medically pointless treatments [19]. However, several relatives that were interviewed for our study actually expressed concerns that physicians were continuing with life-sustaining treatment for too long and that they did not consider the patient’s values and preferences enough.

The questionnaire showed that relatives from the first COVID-19 wave were significantly less likely to be involved in treatment decision-making compared to relatives from pre-COVID-19. There are several possible explanations for this. Firstly, ICUs were faced with an exceptionally high number of patients to care for during the COVID-19 pandemic [20]. Therefore healthcare professionals were likely to have less time to communicate and support relatives, next to the increased provision of medical care. Secondly, due to the visitation restrictions that were in place all communication was done via telecommunication instead of face-to-face. Ramos et al. [14] showed that during the pandemic relatives that had to communicate with healthcare professionals via telecommunication were less satisfied with deliberation regarding decision-making compared to relatives who had face-to-face communication. Thirdly, during the early stages of the pandemic, the course of a COVID-19 infection was quite unpredictable as patients deteriorated rapidly and suddenly and a lot was still unknown about treatment options [21]. Hence, physicians might have had to make abrupt treatment decisions, possibly limiting the possibilities of involvement of relatives. These possible explanations are supported by the finding that the likelihood of involvement in decision-making of relatives from the second COVID-19 wave did not significantly differ from relatives from pre-COVID-19. During the second wave, the number of patients in the ICU was somewhat lower compared to the first wave, face-to-face communication between relatives and ICU healthcare professionals was possible again to some extent, and ICU healthcare professionals generally knew better what to expect regarding the course and treatment of a COVID-19 infection and treatment options were advanced.

### Discordance between actual and preferred role among both relatives preferring a passive and relatives preferring an active role

In line with earlier literature [3–6], relatives have diverse preferences regarding their role in treatment decision-making. While some relatives preferred to have an active role and deliberate with healthcare professionals about the course of (in)action, others preferred to have a more passive role and cede control to the physician. The preference for a more passive role may partially stem from the misconception of relatives that a relative needs to be knowledgeable about the patient’s medical condition and treatment options and that they bear the responsibility of making the decisions. However, as Kon et al. [2] described, the exchange of information is an important pillar of shared decision-making in the ICU. This includes medical information shared by healthcare professionals, but also information about the patient’s values, goals and preferences shared by relatives [2]. Relatives may have more expertise for discussions on treatment decisions than they might think.

In some instances, relatives’ preferences were in discordance with their actual role. Some relatives had a more passive role than they preferred, whereas others were more actively involved than they preferred. Taking into account relatives’ preferences regarding their role in decision-making is of utmost importance since discordance in the preferred and actual role in decision-making can result in increased symptoms of depression and post-traumatic stress disorder [8]. Based on our results we cannot conclude why there discordances between preferred and actual involvement occur, however there are some possible explanations. One of the reasons for this discordance could be that physicians have a tendency to adopt a physician-driven approach, which was also found among Dutch physicians in general [22]. As described earlier this could stem from lack of trust in relatives’ understanding of the situation and fear for more medically pointless treatments [19]. Also, it could be that relatives are unaware of the possibilities of involvement and therefore remain in a more passive role than they prefer. Furthermore, if physicians do not know to what extent relatives wish to be involved discordance between the preferred and actual involvement is more likely to occur, e.g. if a physician adopts a shared decision-making approach while relatives prefer that the physician takes the decision but this is not communicated, a mismatch will occur. Further research is needed to investigate the reasons for discordance. This could be done, for instance, using observational research combined with interviews at multiple moments.

### Discussions on a patient’s values and quality of life as a starting point for involvement in treatment decision-making

As relatives have varying conceptions and preferences regarding involvement in treatment decision-making, discussions about a patient’s values and quality of life could be a good starting point. One of the main reasons for a preference for active involvement in decision-making was to share important information about the patient’s values and quality of life. Relatives in our study also indicated that physicians appreciated relatives sharing this type of personal information and that physicians found it very valuable information. Two studies that analysed audio-recorded family conferences in the ICU showed that in less than half of those conferences patients’ preferences and quality of life were discussed and if so very limited time was devoted to these topics [23, 24]. As the course of a COVID-19 infection was fairly unpredictable during the early months of the pandemic treatment decisions possibly had to be made more ad-hoc than in “usual” ICU practice. Therefore early deliberation with relatives, and patients when still able to communicate, about a patient’s values and quality of life could have been valuable as an underlying basis for the ad-hoc decisions later on in the admission.

Considering the diverse preferences of relatives regarding involvement in treatment decision-making in the ICU and the importance of aligning decisions with a patient’s values and preferences we suggest a two-fold approach: (1) devote a significant amount of time at the start of an ICU admission for discussing with surrogate decision-makers what a patient’s treatment preference would be based on their values and conceptions of quality of life; (2) timely and repeatedly inform relatives about the valuable role they can play in decision-making and discuss with relatives to what extent they can and wish to be further involved in decision-making and tailor the decision-making process accordingly. This may limit the negative impact of discordance in the preferred and actual role in decision-making in relatives as well as prevent conflicts from arising and escalating [25].

### Strengths and limitations

One of the strengths of this study is its mixed-methods design, as the qualitative data from the interviews helped to interpret, explain and further explore the quantitative findings from the questionnaire. This strength was illustrated by the fact that the interviews revealed that relatives had very different conceptions of what involvement in treatment decision-making entailed, which would have remained unknown if only questionnaire data were used. Additionally, in the participating ICUs we have invited all eligible relatives to participate in our study and did not make any selection in this. However, the participating ICUs are all in the Netherlands, and our results might differ to some extent in other countries with different cultures and (healthcare) system.

A limitation of this study is that all interviews were conducted via telephone or video call due to the COVID-19 restrictions. This could have made it more difficult to build rapport with participating relatives, which could in turn have resulted in less in-depth interviews. However, several relatives indicated in the interviews that they appreciated the fact that the interview was not face-to-face, because they felt more comfortable and free to share their experiences via telephone or video call. Also, the data that were used were self-reported by relatives and we do not know to what extend the involvement they reported is the actual involvement that took place. We recommend future researchers to triangulate these kind of self-reported data with observations. Furthermore, for some relatives the ICU admission was relatively long ago when the questionnaire was administered and when the interviews were conducted. This could have led to recall bias. In the analyses of questionnaire data we corrected for the time between ICU admission and questionnaire completion, and in the interviews relatives described very detailed experiences and feelings, suggesting that they were still very well able to recall the ICU admission. Finally, another bias that might be present is non-respondent bias. Analysis showed no difference between responders and non-responders with regards to the gender of the patient and whether the patient had deceased or not, but the kinship to the patient was significantly different.

## Conclusions

The situation in the ICU during the COVID-19 pandemic has learned us more about the involvement of relatives in treatment decision-making and how to address this in normal ICU practice. Relatives of ICU patients are to varying degrees involved in treatment decision-making and have diverse preferences in this regard, which potentially leads to discordance between relatives’ preferred and actual role. Shared decision-making with patients or surrogates is considered a crucial element of care provision, but our results show that this is not yet widely applied in the ICU and that not all relatives prefer this approach. It is very important to align the treatment to a patient’s values and preferences, as well as to align relatives’ preferred and actual role in decision-making. Therefore we suggest that discussions about a patient’s quality of life should be the starting point, followed by tailoring the decision-making process to relatives’ preferences as much as possible. This requires awareness among ICU healthcare professionals that relatives have heterogeneous preferences regarding the treatment decision-making process, which may also change during an ICU admission.

### Electronic supplementary material

Below is the link to the electronic supplementary material.

Supplementary Material 1

Supplementary Material 2

Supplementary Material 3

## Data Availability

The dataset used and/or analysed during the current study is available from the corresponding author upon reasonable request.
