# Supplementary material for: Varying (preferred) levels of involvement in treatment decision-making in the intensive care unit before and during the COVID-19 pandemic: a mixed-methods study among relatives
Source: BMC Med Inform Decis Mak. 2024 Feb 12;24:46. doi: 10.1186/s12911-024-02429-y (PMC10863197; doi:10.1186/s12911-024-02429-y)
Supplement: Supplementary file 1 — Supplementary Material 1 [file 12911_2024_2429_MOESM1_ESM.docx]

**Supplementary file 1. Family support teams (FSTs)**

**Description of Family Support Teams.**

Three ICUs within our sample used one or more Family Support Teams (FSTs) during the first COVID-19 wave from mid-March until mid-May 2020. Most of the key elements of the FSTs were similar among the three ICUs. The FSTs consisted of physicians who were not part of the clinical ICU team and were from a variety of medical specialties: e.g. oncology, anesthesiology and geriatrics. In one ICU, the FSTs also assisted in turning COVID-19 patients from supine to prone position and vice versa. All FSTs worked under authority of the treating physician. The FSTs provided the daily support to relatives via telephone giving primarily clinical updates about the patient and sometimes discussing the well-being of the relative. Critical decisions, such as stopping respiratory support were communicated by the treating physician. FSTs were informed about the situation of the patient via the electronic medical records and in some cases it was possible that they attended multidisciplinary consultation meetings. In one ICU, FST members also attended the daily ward round. One ICU had a psychosocial support team in addition to the FST. This team consisted of spiritual caregivers, medical social workers and psychologists, to whom the FST members could refer if relatives needed additional psychosocial support.

**Differences in involvement in treatment decisions between being supported by the ICU or a FST**

| **Appendix Table 1. Logistic regression: differences in involvement in treatment decisions between being supported by the ICU or a FST (only relatives of the first COVID-19 wave)** | | | | |
| --- | --- | --- | --- | --- |
|  | **Asked to be involved in treatment decisions** | | **Enough time for questions and concerns with treatment decisions** | |
|  | Row %^a^ | Adjusted OR (95% CI)^b^ | Row %^c^ | Adjusted OR (95% CI)^b^ |
| ICU | 64.3 | 1.00 | 83.3 | 1.00 |
| FST | 55.6 | 0.69 (0.29-1.69) | 81.8 | 0.90 (0.21-3.87) |
| ICU = intensive care unit; FST = family support team  ^a^ Percentage of the relatives who reported to have been always, often or occasionally involved in treatment decisions;  ^b^ Adjusted for period between ICU admission and questionnaire completion;  ^c^ Percentage of relatives who reported to have had always or often enough time for questions and concerns with treatment decisions; | | | | |
