# Supplementary material for: Varying (preferred) levels of involvement in treatment decision-making in the intensive care unit before and during the COVID-19 pandemic: a mixed-methods study among relatives
Source: BMC Med Inform Decis Mak. 2024 Feb 12;24:46. doi: 10.1186/s12911-024-02429-y (PMC10863197; doi:10.1186/s12911-024-02429-y)
Supplement: Supplementary file 3 — Supplementary Material 3 [file 12911_2024_2429_MOESM3_ESM.docx]

**Supplementary file 3 – Topic list interview**

- Global description of the ICU admission
- Experiences with the visitation policy
- Experiences with support during the ICU admission
  - Global description of the received support from the hospital
    - Additional support outside the hospital (e.g. general practitioner)
  - Satisfaction with support and important elements
  - Support around the end-of-life (if patient deceased in the ICU)
- Experiences with treatment decision-making during the ICU admission
  - Global description of treatment decision-making
  - Satisfaction with involvement in treatment decision-making
  - Wishes and needs regarding involvement in treatment decision-making
- Experiences with aftercare for relatives (both when a patient was discharged and when a patient deceased in the ICU)
  - Global description of the received aftercare
  - Satisfaction with the aftercare
  - Wishes and needs regarding aftercare
